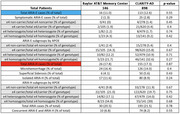# A Retrospective Study of Amyloid‐Related Imaging Abnormalities in Patients Receiving Lecanemab for the Treatment of Alzheimer's Disease

**DOI:** 10.1002/alz70856_099951

**Published:** 2025-12-25

**Authors:** Alexander Andrew Wallace, Oliver Zhou, CIndy Marshall, Claudia Padilla

**Affiliations:** ^1^ Texas A&M College of Medicine, Dallas, TX, USA; ^2^ Baylor University Medical Center, Dallas, TX, USA; ^3^ Baylor AT&T Memory Center, Dallas, TX, USA

## Abstract

**Objective:**

To evaluate the incidence and characteristics of amyloid‐related imaging abnormalities (ARIA) in patients receiving lecanemab for Alzheimer's disease (AD) in routine clinical practice.

**Background:**

ARIA, presenting as brain edema or hemorrhage (ARIA‐E or ARIA‐H), are significant, potentially severe side effects of anti‐amyloid therapies such as lecanemab and pose major safety concerns for patients and clinicians. Therefore, in order for patients and clinicians to make informed decisions about the risks and benefits of lecanemab, an accurate safety profile based on both robust clinical trial and real‐world data must be developed. While the CLARITY‐AD trial demonstrated the overall safety of lecanemab in a clinical trial setting, real‐world safety data of lecanemab remain limited.

**Methods:**

We conducted a retrospective chart review of 146 patients of the Baylor AT&T Memory Center who had initiated treatment with lecanemab for AD between March 1, 2023, and September 30, 2024, and who had completed at least one post‐treatment MRI. Each patient's APOE genotype, presence and evolution of ARIA over time according to MRI impressions, and clinical impressions were assessed and compared to CLARITY‐AD trial data.

**Results:**

Our results closely mirrored those of the CLARITY‐AD trial.^1^ In chi‐squared analysis of our data, there were no statistically significant differences (*p* >0.05) in any of the following measures: the percentages of patients who developed ARIA, ARIA‐E, ARIA‐H, concurrent ARIA‐E and ARIA‐H, microhemorrhages, and superficial siderosis as well as the rates of ARIA‐E, ARIA‐H, and symptomatic ARIA‐E by APOE genotype.

**Conclusions:**

Real‐world data of ARIA incidence, severity, and APOE genotype associations closely match the data obtained in the CLARITY‐AD trial, providing further evidence to support the overall safety of lecanemab in routine clinical practice as guided by appropriate use recommendations.

**References**:

1 ‐ van Dyck CH, Swanson CJ, Aisen P, et al. Lecanemab in Early Alzheimer's Disease. N Engl J Med. 2023;388(1):9‐21. doi:10.1056/NEJMoa2212948